# HEALTH TRAJECTORIES OVER TIME IN THE ATHLOS PROJECT: FINDINGS FROM MULTIPLE COHORTS

**DOI:** 10.1093/geroni/igz038.2939

**Published:** 2019-11-08

**Authors:** Matthew Prina

**Affiliations:** King’s College London, London, United Kingdom

## Abstract

The ATHLOS (Ageing Trajectories of Health: Longitudinal Opportunities and Synergies) project is a consortium of 15 partners across Europe who are working together to understand patterns of healthy ageing trajectories, and to seek the factors that determine those patterns, in a harmonised dataset of 17 international cohort studies of ageing. During this symposium we will be presenting some of the work that has recently been carried out within this project. The symposium will consist of four talks: the first talk will introduce the project, and describe the preliminary work that took place within the first few years of the project, and the challenges faced by the consortium. The second talk will focus on the harmonisation process and on the development of the health metric, an indicator used to measure healthy ageing in this project. The third talk will focus on inequalities in healthy ageing, specifically investigating the impact of education and wealth across cohorts. Finally, in the last talk we will describe the role of lifestyle behaviours (specifically physical activity, smoking and alcohol consumption) and their impact on healthy ageing trajectories.

